# The effect of acute aerobic exercise on arterial stiffness in individuals with different body fat percentages: A cross-sectional study

**DOI:** 10.3389/fcvm.2022.1072191

**Published:** 2023-01-11

**Authors:** Zegui Huang, Guanzhi Chen, Xianxuan Wang, Yiran Zang, Qing Yue, Zefeng Cai, Xiong Ding, Zekai Chen, Zhiwei Cai, Kuangyi Wu, Huancong Zheng, Weiqiang Wu, Shouling Wu, Youren Chen

**Affiliations:** ^1^Shantou University Medical College, Shantou, China; ^2^Department of Cardiology, Second Affiliated Hospital of Shantou University Medical College, Shantou, China; ^3^Second Clinical College, China Medical University, Shenyang, China; ^4^Graduate School, North China University of Science and Technology, Tangshan, China; ^5^School of Public Health, Wuhan University, Wuhan, China; ^6^Department of Epidemiology, University Medical Center Groningen, University of Groningen, Groningen, Netherlands; ^7^Department of Cardiology, Kailuan General Hospital, Tangshan, China

**Keywords:** arterial stiffness, aerobic exercise, acute effect, body fat percentages, arterial compliance

## Abstract

**Background:**

Body fat percentage were positively correlated with arterial stiffness, but the acute change in arterial stiffness after aerobic exercise in individuals with different body fat percentages remains unclear. This study was aimed to determine the effect of acute aerobic exercise on arterial stiffness in individuals with different body fat percentages.

**Methods:**

Individuals who both participated in the seventh survey of the Kailuan study and the fifth iteration of National Physical Fitness Monitoring were enrolled in our study. All participants underwent measurement of brachial–ankle pulse wave velocity, blood pressure, and heart rate before and after a two-stage load test on cycle ergometry. Additionally, the generalized linear model was established to analyse between-group differences of the change in brachial–ankle pulse wave velocity before and after exercise for individuals with different body fat percentages.

**Results:**

The participants (*N* = 940, 36.8 ± 7.7years old, all male) were divided into: Q1 10.0–19.3%, Q2 19.3–23.3%, Q3 23.3–27.1% and Q4 27.1–37.7% by body fat percentage quartile. Overall, after exercise, brachial–ankle pulse wave velocity decreased significantly (before, 1,375.1 ± 209.1; after, 1,341.5 ± 208.0cm/s; *p* < 0.01). After adjusting for confounding factors, the generalized linear model showed that the β values and 95% confidence interval (CI) of Q1, Q2 and Q3 groups were −38.1 (95% CI: −57.3, −19.0), −8.5 (95% CI: −25.8, 3.7),−3.7 (95% CI: −20.5, 13.0), respectively, when compared with Q4. For an increase in body fat percentage by one standard deviation (5.8%), β = 14.5 (95% CI: 7.3, 21.6). Similar results were obtained in sensitivity analyses.

**Conclusions:**

Acute aerobic exercise had a positive effect on the arterial stiffness of adults with different body fat percentages. Compared with individuals with high body fat percentages, the arterial stiffness of people with low body fat percentages had significant reduction after exercise.

## Introduction

Arterial stiffness is a vascular manifestation of aging and an independent risk factor for cardiovascular disease (1, 2). Previous studies have found that, in addition to age, which cannot be changed, lack of exercise, smoking and obesity may accelerate the progression of arterial stiffness (3, 4). Conversely, healthy lifestyle behaviors such as engaging in aerobic exercise, quitting smoking and losing weight can slow or reverse arterial stiffening (5, 6). Although studies have shown that long-term aerobic exercise can reduce arterial stiffness (7), whether the effect of acute aerobic exercise can do the same in different populations has not been confirmed. Siasos et al. found that arterial stiffness immediately decreases after aerobic exercise in healthy young people (8); however, some studies have found that, in people with obesity (9) or diabetes (10), arterial stiffness immediately increases after aerobic exercise.

Previous studies have shown that body mass index (BMI) is an independent predictor of arterial stiffness (4), but recent studies have found that body fat percentages (BF%) reflects body fat content and distribution more accurately than BMI (11) and has a stronger correlation with arterial stiffness (12). In studies with Eastern European white individuals and Indian Asian individuals, BF% were positively correlated with arterial stiffness (12, 13). Nevertheless, the relationship between BF% and acute change in arterial stiffness remains unclear. To our knowledge, no research has explored the acute change in arterial stiffness after aerobic exercise in people with different BF%.

Therefore, we aimed to determine the effect of acute aerobic exercise on arterial stiffness in individuals with different BF% based on data from the Kailuan study and National Physical Fitness Monitoring.

## Methods

### Study design and participants

The Kailuan study is an ongoing longitudinal prospective cohort study in the Kailuan community in Tangshan, China that began in 2006. Since then, follow-ups have been conducted every 2 years, including questionnaires and health assessments. The details of study design and procedures have been described previously (14). To systematically assess the nationwide physical fitness of individuals, National Physical Fitness Monitoring is a project in China that began in 2000 and is conducted every 5 years with sample surveys. In 2020, the fifth iteration of National Physical Fitness Monitoring selected four coal mining companies of Kailuan Group for sampling in Tangshan. Random sampling was used to select 1,200 male employees without cardiovascular disease history between the ages of 20 and 49 years for physical fitness testing.

Among them, 1,138 individuals participated in the seventh survey of the Kailuan study at the same time were included in our study. All participants were asked to perform a two-stage load test on cycle ergometry and undergo brachial–ankle pulse wave velocity (baPWV), blood pressure and heart rate (HR) measurements before and after exercise. We excluded individuals who had not performed the two-stage load test or completed baPWV measurements. The study was conducted in accordance with the principles of the Declaration of Helsinki and was approved by the ethics committee of the Kailuan General Hospital. All participants have provided written informed consent.

### Data collection

The questionnaire survey information (including disease history, medication history, physical exercise, smoking, drinking), anthropometric measurements and pre-exercise biochemical indicators were derived from the seventh follow-up of the Kailuan study. Blood samples were collected from the antecubital vein of participants in the morning after an overnight fast. All biochemical indicators including total cholesterol (TG), triglyceride (TC), fasting blood glucose (FBG), high-density lipoprotein cholesterol (HDL-C), low- density lipoprotein cholesterol (LDL-C), high-sensitivity C-reactive protein (Hs-CRP) and creatinine were measured using an autoanalyzer (Hitachi 747; Hitachi, Tokyo, Japan) at the central laboratory of the Kailuan General Hospital. The estimated glomerular filtration rate (eGFR) was calculated according to the Chronic Kidney Disease Epidemiology Collaboration creatinine equation (15). Anthropometric indices included height, weight and waist circumference. The physician used the inelastic soft ruler to measure the waist circumference of participants to the nearest 0.1 cm. Furthermore, a tape rule was used for measuring the height to the nearest 0.1 cm, and weight was measured to the nearest 0.1 kg by using calibrated platform scales. BMI was measured by body weight (kg) divided by the square of height (m^2^). The BF% and exercise test data were provided by National Physical Fitness Monitoring.

### Body fat percentages measurement

A multi-frequency body composition analyser (GMCS-TZL3, Beijing, China) of the National Fitness Monitoring Center was used to measure the fat mass and BF% of each participant before exercise. All participants were asked to avoid eating, bathing and strenuous activity within the 2 h before the measurement. Participants wore clean thin clothes and stood quietly while barefoot on the electrode pad of the analyser; the hand electrodes were held with both hands and the arms were position at ~15° from the trunk. The measurement lasted ~1–2 min.

### Two-stage load test on cycle ergometry

A cycle ergometry (GMCS-GLC3, Beijing, China) uniformly supported by the National Fitness Monitoring Center was used for the two-stage load test. Participants were asked not to smoke, drink, ingest caffeine or perform vigorous activities within the 12 h before the test. Following a brief warm-up by cycling against unloaded exercise (0 watts) for 30 seconds, and according to the individual's conditions, participants started to cycle at a moderate intensity (50–80 watts) for 3 minutes so that the heart rate reached 60-80% of their estimated maximum (calculated as 220 minus ages in years). Moreover, the load was increased by 25 watts in the next 3 minutes. The last 30 seconds consisted of a zero-load recovery phase. Throughout the exercise, participants needed to maintain a cycling speed of 60 revolutions per minute. Heart rate was monitored in real time with a heart rate monitor worn midlevel on the inner upper right arm, and Maximum oxygen consumption (VO_2_max) was indirectly estimated based on the cycling exercise and calculated by the ergometry, as previously described (16).

### Brachial–ankle pulse wave velocity, heart rate and blood pressure measurements

A networked arteriosclerosis detection device (BP-203RPE III, Omron Health Medical Co., Ltd., China) was used to measure baPWV, HR and blood pressure before and after the two-stage load test. The measurement was performed by two professional personnel. For the pre-exercise measurement, each participant was instructed to lie down on the examination couch in a supine position. Blood pressure cuffs were attached to the upper arms and the ankles. The lower edge of the arm cuff was positioned 2–3 cm above the cubital fossa, while the lower edge of the ankle cuff was positioned 1–2 cm above the medial malleolus. The heart sound collection device was placed in the precordial region of the participants. Electrocardiography electrodes were attached to the left and right wrists. All participants repeat the above measurement after exercise. In our study, both systolic pressure (SBP) and mean arterial pressure (MAP) value using the measurement value of the right ankle artery blood pressure. The baPWV was taken from the larger of the left and right baPWV.

### Relevant definitions

Hypertension was defined as SBP ≥140 mmHg, diastolic blood pressure (DBP) ≥90 mmHg, or (and) having a record of diagnosed hypertension or (and) taking antihypertensive drugs. Diabetes mellitus was defined as FBG ≥7.0 mmol/L, or (and) having a history of diagnosed diabetes or (and) taking hypoglycaemic drugs. Changes in brachial–ankle pulse wave velocity (ΔbaPWV), heart rate(ΔHR), systolic blood pressure (ΔSBP) and mean arterial blood pressure (ΔMAP) were *measurement value after exercise – measurement value before exercise*. Smoking status was defined as smoking lasting more than 1 year, smoking on average ≥1 cigarette/d, and still smoking in the last year. Drinking status was defined as the duration of drinking for more than 1 year, the average drinking ≥ 100ml/d, and the drinking in the last 1 year. Physical exercise was defined as exercise ≥3 times a week, each time duration at least 30min.

### Statistical analysis

The study population was divided into: Q1 10.0–19.3%, Q2 19.3–23.3%, Q3 23.3–27.1% and Q4 27.1–37.7% by BF% quartile. Continuous variables were contrasted by analysis of variance or the Kruskal Wallis test according to distribution, and categoric variables were compared with the chis quare test. Paired-sample *t-*tests were used to compare baPWV, HR and blood pressure values before and after exercise. In addition, the generalized linear model was established to analyse the differences in ΔbaPWV (dependent variable) between groups (i.e., different BF%, independent variable). The model of main analysis was adjusted for age, pre-exercise baPWV, LDL-C, FBG, Hs-CRP, eGRF, interval measurement time, VO_2_max, smoking (yes or no), drinking (yes or no), physical exercise (yes or no), ΔHR and ΔMAP.

Considering for hypertension and diabetes both have an effect on arterial stiffness, Individuals with diabetes mellitus, hypertension or using antihypertensive drugs were excluded to performed the sensitivity analysis, respectively. The SAS 9.4 (SAS Institute, Inc, Cary, NC) software was used for statistical analysis and *p* < 0.05 (two-sided test) was considered statistically significant.

## Results

### Baseline characteristics

**Figure 1:**
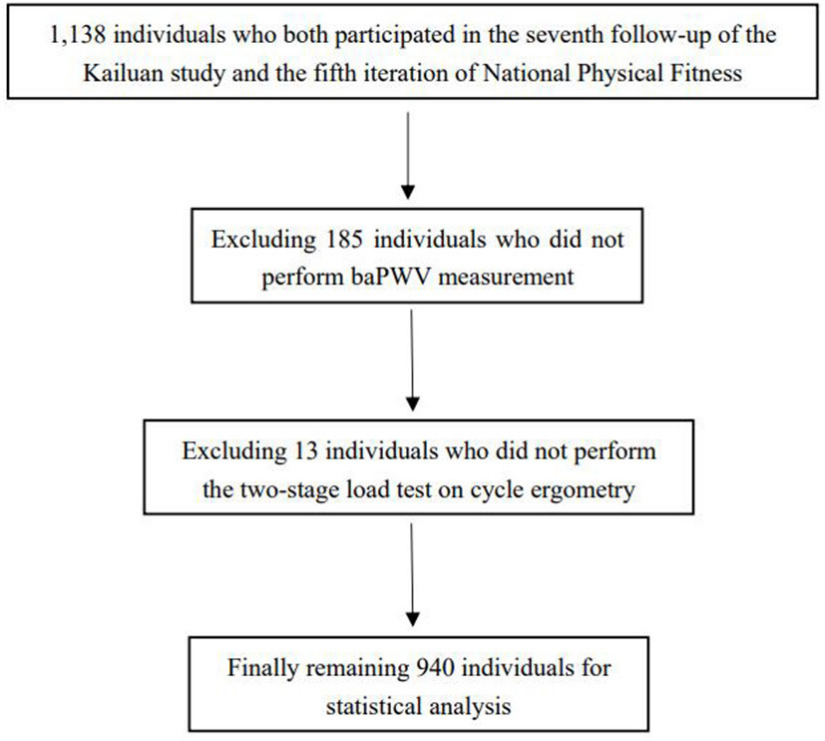
Flow chart of inclusion and exclusion.

A total of 1,138 individuals who both participated in the seventh follow-up of the Kailuan study and the fifth iteration of National Physical Fitness Monitoring. However, 185 individuals did not undergo baPWV measurements and 13 individuals did not perform the two-stage load test were excluded. We finally included 940 individuals (82.6%) in this study's statistical analysis (Figure 1). All participants had a mean age of 36.8 ± 7.7 years old, which all were men, and the mean BF% was 23.1 ± 5.8% (Table 1). Dividing the study population into four group by BF% quartile (i.e., Q1 10.0–19.3%, Q2 19.3–23.3%, Q3 23.3–27.1% and Q4 27.1–37.7%). Compared with the low quantile BF% group, the high quantile BF% group's fat mass, weight, BMI, waist circumference, TG, TC, LDL-C and the proportions of hypertension were higher, but HDL-C was lower, the differences between groups were statistically significant (*p* < 0.05).

**Table 1:** Baseline characteristics for participants with different BF%.

**Variables**	**All** ***N =* 940**	**Q1** ***N =* 235**	**Q2** ***N =* 235**	**Q3** ***N =* 235**	**Q4** ***N =* 235**	**P**
Age (years)	36.8 ± 7.7	36.1 ± 8.1	37.8 ± 7.5	37.3 ± 7.6	36.0 ± 7.6	0.03
Fat mass (kg)	18.2 ± 7.1	9.7 ± 2.6	15.5 ± 1.7	20.1 ± 2.0	27.4 ± 4.7	< 0.01
Weight (kg)	76.3 ± 12.4	63.1 ± 6.3	72.1 ± 5.9	79.3 ± 6.2	90.7 ± 9.9	< 0.01
BF%	23.1 ± 5.8	15.1 ± 3.1	21.5 ± 1.1	25.3 ± 1.0	30.0 ± 2.2	< 0.01
BMI (kg/m^2^)	26.2 ± 3.8	21.8 ± 2.0	25.1 ± 1.8	27.3 ± 1.9	30.7 ± 2.8	< 0.01
WC (cm)	89.8 ± 9.7	78.7 ± 6.1	87.1 ± 5.4	92.6 ± 4.6	100.2 ± 6.7	< 0.01
TG (mmol/L)	1.2 (0.8, 1.9)	0.9 (0.6, 1.2)	1.1(0.8, 1.7)	1.3 (0.9, 2.1)	1.6(1.0, 2.3)	< 0.01
TC (mmol/L)	4.7 ± 1.1	4.5 ± 1.3	4.8 ± 1.0	4.8 ± 0.9	4.8 ± 1.0	< 0.01
HDL-C (mmol/L)	1.3 ± 0.3	1.4 ± 0.3	1.3 ± 0.2	1.3 ± 0.3	1.2 ± 0.4	< 0.01
LDL-C (mmol/L)	2.8 ± 0.8	2.5 ± 0.6	2.8 ± 1.0	2.8 ± 0.7	2.9 ± 0.7	< 0.01
FBG (mmol/L)	5.5 ± 0.9	5.4 ± 0.7	5.5 ± 1.1	5.6 ± 1.1	5.5 ± 0.7	0.22
hs-CRP (mmol/L)	0.3(0.0, 1.5)	0.3(0.1, 1.5)	0.3(0.0, 1.4)	0.2(0.0, 1.2)	0.4(0.0, 2.0)	0.01
eGFR (ml/min/1.73 m^2^)	106.9 ± 24.9	108.7 ± 38.9	104.3 ± 16.7	107.1 ± 21.8	107.3 ± 15.3	< 0.01
Diabetes mellitus n (%)	66 (7.0)	15 (6.3)	11 (4.6)	24 (10.2)	16 (6.8)	0.12
Hypertension n (%)	319 (33.9)	51(21.7)	69 (29.3)	99 (42.1)	100 (42.5)	< 0.01
Antihypertensive drugs n (%)	94 (10.0)	11 (4.6)	22 (9.3)	32 (13.6)	29 (12.3)	< 0.01
Physical exercise n (%)	352 (37.4)	84 (35.7)	89 (37.8)	94 (40.0)	85 (36.1)	0.77
Smoking n (%)	554 (58.9)	141 (60.0)	137 (58.3)	136 (57.8)	140 (59.5)	0.96
Drinking n (%)	523 (55.6)	123 (52.3)	131 (55.7)	134 (57.0)	135 (57.4)	0.67

Data are presented as mean ± SD, n (%), or median (interquartile range). body fat percentage quartiles: Q1:10.0–19.3%; Q2: 19.3–23.3%; Q3: 23.3~27.1%; Q4: 27.1~37.7%; BF%, body fat percentage; BMI, body mass index; WC, waist circumference; HDL-C, high-density lipoprotein cholesterol; LDL-C, low- density lipoprotein cholesterol; TC, total cholesterol; TG, Triglyceride; FBG, fasting blood glucose; hs-CRP, high-sensitivity C-reactive protein; eGFR, estimated glomerular filtration rate.

### Brachial–ankle pulse wave velocity, heart rate, blood pressure before and after exercise

Compared with the low quantile BF% group, the high quantile BF% groups had higher baPWV, HR, SBP and MAP before exercise (Table 2). After exercise, the baPWV of participants significantly decreased (before, 1,375.1 ± 209.1; after, 1,341.5 ± 208.0 cm/s; *p* < 0.01), while their HR increased (before, 80.2 ± 12.5; after, 85.7 ± 13.5bpm; *p* < 0.05). Additionally, significant changes have observed in SBP or MAP of participants before and after exercise. Compared with individuals who are high BF%, the individuals with low BF% have higher VO_2_max, and the between-group difference were statistically significant (*p* < 0.05), while the between-group difference for peak heart rate were not statistically significant.

**Table 2:** Pre-exercise and post-exercise arterial stiffness and vessel hemodynamic parameters for participants with different BF%.

**Variables**	**All** ***N =* 940**	**Q1** ***N =* 235**	**Q2** ***N =* 235**	**Q3** ***N =* 235**	**Q4** ***N =* 235**	**P**
Pre-exercise HR (bpm)	80.2 ± 12.5	77.6 ± 12.4	79.8 ± 11.5	80.6 ± 12.4	82.8 ± 13.1	< 0.01
Post-exercise HR (bpm)	85.7 ± 13.5^*^	84.1 ± 14.7^*^	84.9 ± 12.5^*^	86.3 ± 13.5^*^	87.5 ± 13.2^*^	0.03
ΔHR (bpm)	5.0 (0.0,10.0)	5.0 (0.0,12.0)	5.0 (0.0,10.0)	5.0 (0.0,11.0)	4.0 (−1.0,9.0)	0.24
Pre-exercise SBP (mmHg)	141.8 ± 19.8	133.4 ± 16.7	140.4 ± 17.6	145.3 ± 21.6	148.1 ± 19.7	< 0.01
Post-exercise SBP (mmHg)	138.7 ± 20.1^*^	129.5 ± 17.1^*^	137.1 ± 19.1^*^	143.2 ± 20.2^*^	144.8 ± 20.0^*^	< 0.01
ΔSBP (mmHg)	−3.0 (−10.3,4.3)	−3.9 (−10.3,2.5)	−3.7 (−10.3,4.9)	−1.5 (−10.3,5.7)	−3.1 (−11.1,4.1)	0.22
Pre-exercise MAP (mmHg)	96.5 ± 12.7	90.6 ± 11.4	95.3 ± 11.6	99.2 ± 13.1	101.1 ± 12.9	< 0.01
Post-exercise MAP (mmHg)	95.2 ± 13.1^*^	88.9 ± 11.5^*^	93.9 ± 12.9^*^	98.1 ± 12.6^*^	99.2 ± 12.5^*^	< 0.01
ΔMAP (mmHg)	−0.9 (−5.6,3.5)	−2.1 (−5.7,2.5)	−0.2 (−5.7,4.1)	−0.1 (−5.0,4.2)	−1.1 (−5.9,3.5)	0.25
Pre-exercise baPWV (cm/s)	1,375.1 ± 209.1	1,325.1 ± 201.9	1,361.7 ± 182.0	1,419.5 ± 226.2	1,393.9 ± 212.8	< 0.01
Post-exercise baPWV (cm/s)	1,341.5 ± 208.0^*^	1,280.4 ± 195.8^*^	1,335.1 ± 180.8^*^	1,391.7 ± 228.5^*^	1,358.7 ± 209.2^*^	< 0.01
ΔbaPWV (cm/s)	−36.0 (−88.0,24.0)	−43.0 (−89.0,13.0)	−28.0 (−83.0,35.0)	−31.0 (−85.0,32.0)	−39.0 (−92.0,12.0)	0.20
Time (min)	7.1 (4.9,11.1)	7.2 (4.9,11.2)	6.5 (4.9,10.1)	7.7 (5.0,11.7)	6.9 (4.8,11.3)	0.28
VO_2_max (ml/kg/min)	42.5 ± 8.9	49.1 ± 10.0	43.2 ± 6.5	41.2 ± 7.6	36.7 ± 6.2	< 0.01
Peak heart rate (bpm)	139.3 ± 14.4	140.6 ± 15.3	139.4 ± 14.0	138.4 ± 14.2	138.9 ± 14.0	0.38

Data are presented as mean ± SD, median (interquartile range). body fat percentage quartiles:Q1:10.0–19.3%; Q2:19.3–23.3%; Q3:23.36–27.1%; Q4:27.1–37.7%; SBP, systolic blood pressure; DBP, diastolic blood pressure; MAP, mean arterial pressure; HR, heart rate; baPWV, brachial-ankle pulse wave velocity; Time, interval measurement time; VO_2_max, maximum oxygen consumption; ΔHR/ΔSBP/ΔMAP/ΔbaPWV, changes in brachial–ankle pulse wave velocity, heart rate, systolic blood pressure and mean arterial blood pressure.^*^p < 0.05, compared with pre-exercise values.

### Generalized linear analysis results

In model of main analysis (Table 3), after adjusting for confounding factors, with the Q4 group as the control, the β values and 95% confidence interval (CI) of Q1, Q2 and Q3 groups were −38.1 (95% CI: −57.3, −19.0), −8.5 (95% CI: −25.8, 3.7), and−3.7 (95% CI: −20.5, 13.0), respectively. And for an increase in BF% by one standard deviation (5.8%), β = 14.5 (95% CI: 7.3, 21.6).

**Table 3:** Generalized linear analysis results of the between-group difference of ΔbaPWV with BF%.

	**BF%**	**β**	**SE**	**T**	**P**	**95%CI**
Main analysis	Q4 (27.1~37.7)	Ref				
	Q1 (10.0~19.3)	−38.1	9.7	−3.9	0.001	−57.3, −19.0
	Q2 (19.3~23.3)	−8.5	8.8	−0.9	0.331	−25.8, 8.7
	Q3 (23.3~27.1)	−3.7	8.5	−0.4	0.660	−20.5, 13.0
	Per SD	14.5	3.6	3.9	0.001	7.3, 21.6

The model was adjusted for pre-exercise baPWV, age, LDL, FBG, hs-CRP, eGFR, VO_2_max, interval measurement time, smoking, drinking, physical, ΔHR and ΔMAP in main analysis. Per SD, per standard deviation (5.8%) change in BF%.

### Sensitivity analysis

The results of the sensitivity analysis (Table 4) of excluding individuals with diabetes mellitus, hypertension or using antihypertensive drugs, respectively, were consistent with the those of the main analysis, which indicated the main results were robust.

**Table 4:** Sensitivity analysis: Generalized linear analysis results of the between-group difference of ΔbaPWV with BF%.

	**BF%**	**β**	**SE**	***T***	**P**	**95%CI**
Sensitivity analysis 1	Q4 (27.1–37.7)	Ref				
	Q1 (10.0–19.3)	−35.1	9.9	−3.5	0.001	−54.6, −15.5
	Q2 (19.3–23.3)	−8.8	8.9	−1.0	0.321	−26.4, 8.6
	Q3 (23.3–27.1)	−7.2	8.8	−0.8	0.412	−24.5, 10.1
Sensitivity analysis 2	Q4 (27.1–37.7)	Ref				
	Q1 (10.0–19.3)	−44.8	12.0	−3.7	0.001	−68.4, −21.2
	Q2 (19.3–23.3)	−14.4	10.8	−1.3	0.183	−35.8, 6.8
	Q3 (23.3–27.1)	−8.4	11.1	−0.7	0.477	−30.1, 13.3
Sensitivity analysis 3	Q4 (27.1–37.7)	Ref				
	Q1 (10.0–19.3)	−35.1	10.1	−3.5	0.001	−54.9, −15.2
	Q2 (19.3–23.3)	−8.8	9.1	−1.0	0.330	−26.8, 9.0
	Q3 (23.3–27.1)	0.6	8.9	0.1	0.945	−17.0, 18.2

Sensitivity analysis 1 excluded people with diabetes(N = 66), Sensitivity analysis 2 excluded people with hypertension (N = 319), Sensitivity analysis 3 excluded people taking anti-hypertensive drugs (N = 94), adjusted covariates are the same as model in the main analysis.

## Discussion

To the best of our knowledge, our study is the first large-sample study to investigate acute changes in arterial stiffness before and after aerobic exercise in adults with different BF%. The most important finding of our study was that aerobic exercise can immediately reduce arterial stiffness in individuals with different BF%. Compared with people with high BF%, the arterial stiffness of people with low BF% reduced significantly more after exercise.

Current findings on the effect of acute aerobic exercise on arterial stiffness indifferent populations were not consistent. In some studies, aerobic exercise appears to acutely improve arterial stiffness; in a study with African American (*N* = 27, age: 25 ± 4 years old) and white (*N* = 35, age: 24 ± 4 years old) healthy individuals, Schroeder et al. found that carotid–femoral pulse wave velocity (cfPWV) decreased immediately after maximum aerobic exercise (8). Similarly, Chuensiri et al. found that among obese (*N* = 17, BMI: 25.4 ± 0.8 kg/m^2^) and non-obese or overweight (*N* = 18, BMI: 17.1 ± 0.7 kg/m^2^) prepubescent boys, baPWV decreased significantly after intermittent high-intensity aerobic exercise at 130% and 170% of VO_2_max (17). These findings are consistent with those from our study with people with different BF%, that is, aerobic exercise can immediately reduce arterial stiffness.

However, some study held the view that aerobic exercise does not appear to acutely reduce arterial stiffness in obese adult. For instance, in a study with white individuals in the United States (*N* = 67, age: 24 ± 1 years), Kankan et al. found that non-obese people (*N* = 46, BMI < 25 kg/m^2^) exhibited decreased cfPWV after aerobic exercise, while obese people (*N* = 21, BMI>30 kg/m^2^) exhibited increased cfPWV (9). Moreover, the results of a meta-analysis showed that in middle-age and older adult people who are obese, aerobic exercise usually does not improve their arterial stiffness (18). These conclusions are inconsistent with those from our study's findings, which may be due to the small sample size and uncertainty of the results in the above-mentioned studies. Additionally, the difference in the type of pulse wave velocity could be another reason. A meta-analysis reported that arterial stiffness in the peripheral segments was significantly reduced, but it has not significantly reduced arterial stiffness in the central segment after aerobic exercise (19). In our study, the arterial stiffness of individuals with high BF%, as measured by baPWV, decreased after aerobic exercise, but the degree of reduction was not as great as that of people with low BF%. The effect of acute aerobic exercise on blood vessels depends largely on factors such as physiological age, type of exercise (20), exercise intensity (21) and time spent in physical activity. Furthermore, decreasing in baPWV also depend on the cardiorespiratory condition, the participants in our study were mainly young and middle-age men, and we found the individuals with low BF% have higher VO_2_max than those who have high BF%. Therefore, aerobic exercise of longer duration or higher intensity may be required to further decrease arterial stiffness in people with high BF%.

The mechanism of the effect of acute aerobic exercise on arterial stiffness in individuals with different BF% remained still unclear. In our study, all participants underwent only one 7-min duration aerobic exercise session, which may affect arterial stiffness functionally, but would not cause changes to vascular structure. Matthew et al. observed a decrease in insulin resistance and pro-inflammatory state-related metabolites, an increase in the bioavailability of nitric oxide and an increase in fat browning–related metabolites and enhanced lipolysis after a single aerobic exercise session (22). Changes in the levels of these metabolites lead to enhanced vasodilation function and reduce arterial stiffness immediately after aerobic exercise. Moreover, some studies have shown that short-term vigorous exercise will increase shear stress within blood vessel walls as a result of forces exerted by contracting muscles, increase blood flow, improve the expansion and compliance of the artery and reduce the stiffness of the artery (23, 24). We found that, compared with people with high BF%, the arterial stiffness of people with low BF% reduced significantly after aerobic exercise. Therefore, we suppose that, in people with high BF%, beneficial metabolites and blood flow changes may be weaken and indicate that glucose and lipid metabolism disorders or the squeezing of blood vessels by adipose tissue may limit the vascular benefits of exercise.

Our study has some clinical significance. At present, the global population with obesity is rising sharply, and eating foods that are rich in high-fat and highly refined carbohydrates and sedentary lifestyles are the main reasons for the excessive accumulation of body fat (25). The World Health Organization recommends the definition of obesity as male BF%≥25% or female BF% ≥35% (26). High BF% is the risk factor of arterial stiffness, but aerobic exercise can prevent from progressing of arterial stiffness. We found that acute aerobic exercise had different effects on arterial stiffness in people with different BF%. Individuals with low BF% directly benefit from aerobic exercise, but those who with high BF% need to improve their diet habits, lose weight and engage in mid-to-high intensity aerobic exercise to reduce arterial stiffness better (27, 28).

The main strength of this study was the large sample size—over 900 people—which increases the reliability of the results. Previous studies on the acute effects of aerobic exercise on arterial stiffness usually have had sample sizes that ranged from a dozen to dozens (8, 9, 16). An additional strength was that we innovatively established generalized linear model to analyse ΔbaPWV and BF% (with adjustments for confounding factors), which increase the accuracy of the results. Unavoidably, This study has some limitations. First, the baPWV was using as a measurement of arterial stiffness instead of carotid–femoral pulse wave velocity, which is the gold standard of assessing the aorta (29); but the validity and accuracy of baPWV against carotid–femoral pulse wave velocity have been demonstrated in previous studies (30). Second, in this study, bioelectrical impedance analysis (BIA) was used to measure BF%. Dual-energy X-ray absorptiometry (DXA) is the gold standard for measuring human body composition; however, many clinical experiments have confirmed that BIA and DXA are highly correlated and consistent in measuring body fat mass and BF% (31, 32). In addition, BIA imposes no radiation hazard on the human body, is convenient and is suitable for large-sample epidemiological research (33). Third, the generalizability of the results is relatively limited because our participants were young and middle-age men engaged in coal mining in northern China and not included woman. Finally, because of space and personnel constraints, we only performed one baPWV measurement on participants after exercise rather than multiple measurement. Thus, it was difficult to know how long the effect of acute aerobic exercise on arterial stiffness lasted.

## Conclusion

Arterial stiffness in people with different BF% reduce immediately after aerobic exercise, but it may be short-term change. Compared with people with high BF%, the arterial stiffness of people with low BF% significantly reduced after aerobic exercise.

## Data availability statement

The raw data supporting the conclusions of this article will be made available by the authors, without undue reservation.

## Ethics statement

The studies involving human participants were reviewed and approved by the Ethics Committee of Kailuan General Hospital. The patients/participants provided their written informed consent to participate in this study.

## Author contributions

ZH, GC, SW, and YC designed the study idea. ZH, GC, XW, YZ, QY, ZefC, XD, ZekC, and ZhiC analyzed and interpreted the data. ZH, GC, KW, HZ, and WW were responsible for drafting the manuscript. SW and YC reviewed the manuscript. All authors have read and approved the final manuscript.
